# Oxygen metabolism analysis of a single organoid for non-invasive discrimination of cancer subpopulations with different growth capabilities

**DOI:** 10.3389/fbioe.2023.1184325

**Published:** 2023-05-18

**Authors:** Yuji Nashimoto, Shotaro Shishido, Kunishige Onuma, Kosuke Ino, Masahiro Inoue, Hitoshi Shiku

**Affiliations:** ^1^ Institute of Bioengineering and Biomaterials (IBB), Tokyo Medical and Dental University (TMDU), Tokyo, Japan; ^2^ Frontier Research Institute for Interdisciplinary Sciences (FRIS), Tohoku University, Sendai, Miyagi, Japan; ^3^ Graduate School of Environmental Studies, Tohoku University, Sendai, Miyagi, Japan; ^4^ Graduate School of Engineering, Tohoku University, Sendai, Miyagi, Japan; ^5^ Graduate School of Medicine, Kyoto University, Kyoto, Japan

**Keywords:** scanning electrochemical microscopy (SECM), intratumor heterogeneity (ITH), patient-derived cancer organoids, oxygen metabolism, drug screening platform

## Abstract

Heterogeneous nature is a pivotal aspect of cancer, rendering treatment problematic and frequently resulting in recurrence. Therefore, advanced techniques for identifying subpopulations of a tumour in an intact state are essential to develop novel screening platforms that can reveal differences in treatment response among subpopulations. Herein, we conducted a non-invasive analysis of oxygen metabolism on multiple subpopulations of patient-derived organoids, examining its potential utility for non-destructive identification of subpopulations. We utilised scanning electrochemical microscopy (SECM) for non-invasive analysis of oxygen metabolism. As models of tumours with heterogeneous subpopulations, we used patient-derived cancer organoids with a distinct growth potential established using the cancer tissue-originated spheroid methodology. Scanning electrochemical microscopy measurements enabled the analysis of the oxygen consumption rate (OCR) for individual organoids as small as 100 µm in diameter and could detect the heterogeneity amongst studied subpopulations, which was not observed in conventional colorectal cancer cell lines. Furthermore, our oxygen metabolism analysis of pre-isolated subpopulations with a slow growth potential revealed that oxygen consumption rate may reflect differences in the growth rate of organoids. Although the proposed technique currently lacks single-cell level sensitivity, the variability of oxygen metabolism across tumour subpopulations is expected to serve as an important indicator for the discrimination of tumour subpopulations and construction of novel drug screening platforms in the future.

## 1 Introduction

Recent advances in our understanding of the molecular mechanisms of tumours have enabled the development of novel therapeutic modalities targeting specific oncogenic signalling pathways (Koboldt et al., 2012; Bagchi et al., 2021). However, even after the state-of-the-art treatments, most advanced cancers eventually become resistant, leading to recurrence (Holohan et al., 2013; Jenkins et al., 2018). This resistance is thought to arise from pre-existing intratumor heterogeneity (ITH) and plasticity (Swanton, 2012; McGranahan and Swanton, 2017). Therefore, gaining a deeper understanding of ITH and the nature of heterogenous tumour subpopulations can offer novel insights to tackle the issue of therapeutic resistance of cancer.

Organoid technology is promising for evaluating ITH because it preserves the original molecular and histological tumour characteristics (Kondo et al., 2011; van de Wetering et al., 2015; Sachs et al., 2018; Tamura et al., 2018). Recent studies have demonstrated that for numerous pharmaceuticals, the *in vitro* response of patient-derived organoids could reflect the clinical reaction of primary tumours (Weeber et al., 2017; Kondo et al., 2019; Kondo and Inoue, 2019). The high clinical accuracy of patient-derived organoids has expedited the development of organoid repositories and their utilisation in pharmacogenomics and high-throughput screening analyses.

Recently, our research group revealed that colorectal cancer (CRC) cells are composed of distinct but interchangeable subpopulations (Coppo et al., 2023). Some isolated CRC cells generate small spheroids (S-cells), whereas the others produce large spheroids (L-cells). The S-cells give rise to small spheroids composed exclusively of S-cells, whereas the L-cells generate larger spheroids composed of both S- and L-cells. Notably, spheroids composed of S-cells exhibit high resistance to chemotherapy, potentially representing an important subpopulation in drug resistance. Thus, S-cells are a valuable resource for evaluating drug resistance *in vitro*. Unfortunately, the S- and L-cells in patient tumours or organoids can only be discriminated at 2–3 weeks after the initiation of single-cell culture but not at early stages, rendering them impracticable for drug screening purposes. Consequently, a quantifiable sorting technique is warranted to apply the S- and L-cells as drug-screening materials.

The sorting technique for drug screening should not involve any reagents or steps that may harm the samples. Electrochemical techniques utilising a probe-type (Shiku et al., 2001) or patterned (Hiramoto et al., 2017; Kanno et al., 2017) electrode or combining an electroluminescence method (Iwama et al., 2020; Hiramoto et al., 2021) are suitable in this context. In majority of the electrochemical techniques, the oxygen metabolism of a sample is evaluated by analysing the reduction current of oxygen in its vicinity. Of note, when an appropriate electrode is selected, the product of the oxygen reduction reaction is limited to H_2_O, and no harmful materials are therefore involved in the assessment. In particular, scanning electrochemical microscopy (SECM) is the most reliable method for evaluating the oxygen metabolism of biological samples, including human embryos (Kurosawa et al., 2016) and islets (Goto et al., 2009), and it is commercially accessible. For the application of SECM to cancer cells, utilizing the non-invasive feature of SECM, we previously evaluated the longitudinal impact of drugs on cell death (Torisawa et al., 2005a; Torisawa et al., 2005b), formation of necrotic core (Mukomoto et al., 2020), and metabolic changes following drug administration through engineered vasculature (Nashimoto et al., 2023).

**FIGURE 1:**
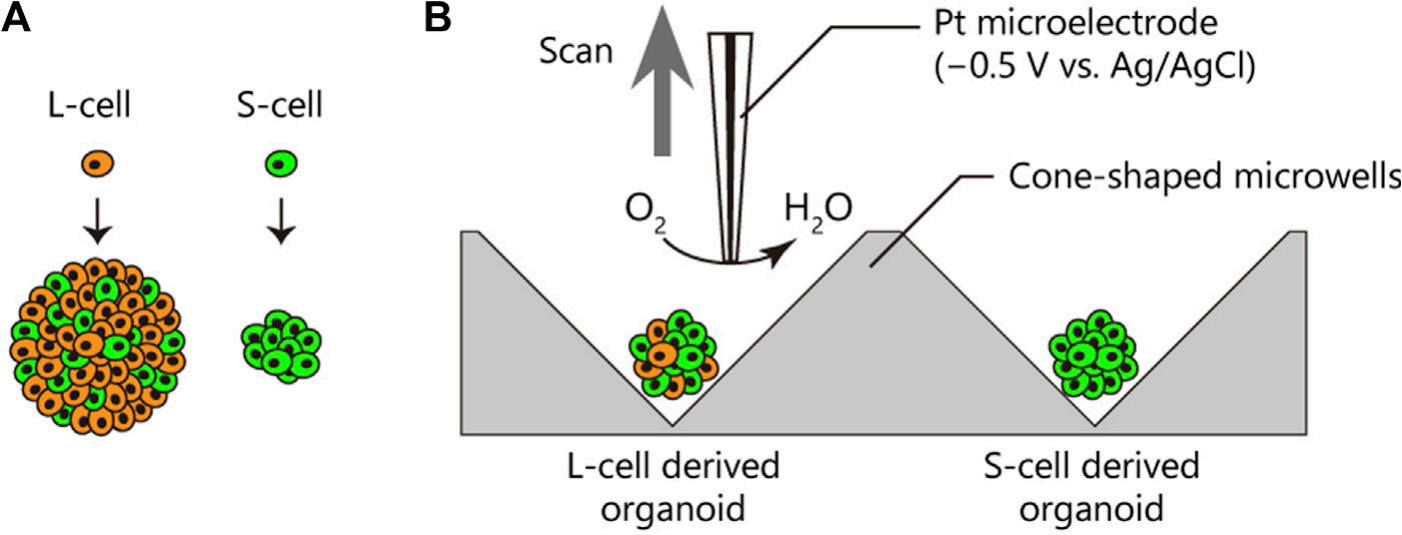
Oxygen consumption rates (OCRs) of patient-derived cancer organoids from different subpopulations. **(A)** Cancer cells in the patient-derived cancer organoid comprised two distinct subpopulations, namely, L-cells (orange) and S-cells (green), generating large and small organoids, respectively (Coppo et al., 2023). L-cells generated a heterogeneous organoid comprising L- and S-cells. **(B)** OCRs of cancer organoids derived from L- and S-cells were compared using the electrochemical method.

To explore potential indicators for discriminating S-cells and L-cells non-invasively, in the present study, we evaluated and compared the oxygen metabolism of patient-derived organoids generated from S- and L-cells using SECM (Figure 1). Our results confirmed the feasibility of applying SECM to discern heterogeneous oxygen metabolism in patient-derived cancer organoids, a phenomenon that is not discernible in conventional cancer cell lines. Moreover, our findings indicated that the L-cell-derived organoids displayed higher oxygen than the S-cell-derived organoids. Although further examination is warranted, our results and applicability of SECM are promising for developing drug screening platforms utilising S-cells for cancer.

## 2 Materials and methods

### 2.1 Cell culture

CRC-derived organoids (KUC16) were generated using the cancer tissue-originated spheroid (CTOS) methodology (Kondo et al., 2011). The organoids were maintained in a non-treated multi-well plate containing the specialised StemPro™ hESC SFM (Thermo Fisher Scientific, United States) organoid medium with 100 U/mL Penicillin/streptomycin (P/S, Thermo Fisher Scientific). For passaging, the organoids were subjected to chemical and mechanical disruption. Briefly, organoids were rinsed with Hanks balanced salt solution (HBSS, Thermo Fisher Scientific) before treatment with TrypLE Select (Thermo Fisher Scientific) at 37°C for 8 min. Thereafter, the organoids were fragmented into smaller clusters via pipette-assisted mechanical disruption (10–100 µL tip, 7–10 strokes) in organoid medium with 10 µM CultureSure Y-27632 (Wako, Japan). The next day, the medium was replaced with organoid medium without Y-27632. Organoids spontaneously reformed from these small clusters. To replace the medium, the whole medium containing the organoids was collected in a conical tube and centrifuged at 400 g for 3 min at room temperature. The organoid medium was replaced every 3 days. When the diameters of the organoids reached 100–300 μm, typically within 2–8 days, the organoids were employed for the analysis of oxygen metabolism.

Subclones of C45 (designated C45-1 and C45-4L) were established as previously described (Coppo et al., 2023). Briefly, C45-1 was derived from a specific subpopulation of C45 organoids exhibiting small diameters using the single-cell-derived spheroid forming and growth (SSFG) assay. C45-4L was established through four rounds of collection and SSFG assay from a subpopulation of C45 with large diameters. Notably, C45-1 showed limited growth potential and was composed solely of S-cells, whereas C45-4L showed substantial growth potential and was composed of a heterogeneous mixture of S- and L-cells. Both C45-1 and C45-4L were amenable to cryopreservation. C45-1 and C45-4L utilised in the present study were obtained from cryogenic stocks. Details are available elsewhere (Coppo et al., 2023). The growth rates of C45-1 and C45-4L were assessed after thawing. Briefly, 5 µL of cell suspension (50 organoids/µL) in 100% Matrigel was dispensed at three sites in a 24-well plate (Iwaki, Japan; 15 µL/well in total) and overlaid with 1 mL of organoid medium containing 10 µM CultureSure Y-27632. The medium was replaced with a regular organoid medium without Y-27632 a day after thawing. The growth of C45-1 and C45-4L was monitored for 1–4 days after thawing.

All subclones of C45 were maintained in the organoid medium supplemented with 2% Matrigel growth factor reduced (Corning, United States). The organoid medium with 2% Matrigel was replaced every 3 days. When the diameters of C45-4L and C45-1 reached 200 μm, typically within 2–4 days, the organoids were employed for the analysis of oxygen metabolism. Before electrochemical oxygen metabolism analysis, organoids were treated with 2 mg/mL collagenase IV (Thermo Fisher Scientific) at 37°C for 1 h to digest Matrigel around the organoids. The institutional ethics committees of the Osaka International Cancer Institute (1803125402), Kyoto University (R2537), and Tohoku University (20A-2) approved the study.

The RIKEN BRC provided HCT116 cells through the National Bio-Resource Project of the MEXT/AMED, Japan. HT29 cells were obtained from the American Type Culture Collection (ATCC). Owing to their widespread usage as CRC cell lines, HCT116 and HT29 were utilised in the present study as controls to contrast patient-derived CRC organoids in terms of oxygen metabolism. HCT116 and HT29 cells were cultured in low-glucose DMEM (Thermo Fisher Scientific) supplemented with 10% foetal bovine serum (FBS, Thermo Fisher Scientific) and 250 U/mL P/S. To dissociate HCT116 and HT29, 0.25% trypsin–EDTA (Thermo Fisher Scientific) was used. Following passage, the culture medium was refreshed every 3 days. The frequency of passages was around once a week. For spheroid formation from HCT116 and HT29 cells, a 20 µL droplet containing ∼150 cells/drop was hung on the lid of a 35-mm dish (Corning) and cultured for 2 days in the drop. All cells were maintained in a humidified incubator at 37°C under 5% CO_2_.

### 2.2 Probe fabrication and electrochemical measurement system

All measurements for the analysis of oxygen metabolism were performed using the HV-405 SECM system (Hokuto Denko, Japan) (Shiku et al., 2004). Briefly, organoids were individually transferred into a plate equipped with six cone-shaped microwells (Clino, Japan) containing 5 mL of measurement solution (ERAM-2, Research Institute for the Functional Peptides, Japan). Once the organoid settles to the bottom of the well, a difference in oxygen concentration is formed due to respiration by the organoid. Ag/AgCl was used as the reference and counter electrodes. A Pt microdisk electrode (diameter = 1–10 µm) sealed with a tapered soft-glass capillary (World Precision Instruments, United States) was fabricated as described previously (Shiku et al., 2001) and used as a working electrode. The Pt electrode was subjected to a potential of −0.5 V vs. Ag/AgCl, and the oxygen reduction current was monitored. The Pt electrode was meticulously positioned close to the organoid under observation with an inverted optical microscope during potential application using an XYZ manipulator. It was scanned vertically up to 500 μm, enabling continuous measurement of the change in oxygen reduction current from the proximity of the organoid to the bulk.

The mass transfer rate of a sample can be determined through the application of SECM and spherical diffusion theory (Shiku et al., 2004). Briefly, oxygen concentration near the spheroid, *C*, was calculated according to the following equation:

$$C=\frac{\left(C_{s}-C^{*}\right)}{L_{p}}r_{s}+C^{*}$$

where, *r*_*s*_ is the radius of the spherical sample, *L*_*p*_ is the distance from the cone tip, and *C*_*s*_ and *C* ^*∗*^ are oxygen concentrations at the surface of the sample and in the bulk (0.21 mol/m^3^), respectively.

Notably, in this method, the system can be self-calibrated at every scan, as oxygen reduction current at the bulk, where oxygen concentration remains near constant, is utilised. To obtain an oxygen reduction current profile, we placed an electrode adjacent to the spheroids or organoids and scanned it vertically employing the side-scanning technique. In the scanning mode, *L*_*p*_ can be expressed as follows:

$$L_{p}={\left\{{\left(h+z\right)}^{2}+r_{s}^{2}\right\}}^{0.5}$$

$$h=\frac{r_{s}}{\sin\theta }$$

where *θ* is the cone’s half-angle (=45° in the present study), and *z* is a scanning distance from the side of the sample.

Then, the oxygen consumption rate [*OCR* (mol/s)] can be obtained from ∆*C* (= *C* ^*^ – *C*_*s*_), as follows:

$$OCR=\frac{D}{0.7}\times 2\pi \left(1-\frac{1}{\sqrt{2}}\right)\left(1+\sqrt{2}\right)R∆C$$

where *D* is the diffusion coefficient of oxygen (2.1 × 10^−9^ m^2^/s).

To eliminate the size effect, we evaluated OCR per sample volume (OCR/V).

### 2.3 Image acquisition and statistical analysis

All optical images were acquired using an inverted microscope (ECLIPSE Ts2, Nikon, Japan) equipped with a CMOS camera (Michrome 20, BioTools, Japan) and analysed using ImageJ (National Institutes of Health, United States). Dimensions of organoids and spheroids were measured from the optical image using Fiji (Schindelin et al., 2012). The contour of each structure was approximated by an ellipse, and diameter was calculated as the average of its major and minor axes.

Statistical analysis was performed, and all boxplots were generated using RStudio. Significance was assessed using a non-parametric Mann–Whitney *U*-test for single comparisons and using non-parametric factorial ANOVA for align-and-rank data. The results were considered significant at *p* < 0.05.

## 3 Results and discussion

### 3.1 Comparison of OCR amongst cancer organoids and cell lines

**FIGURE 2:**
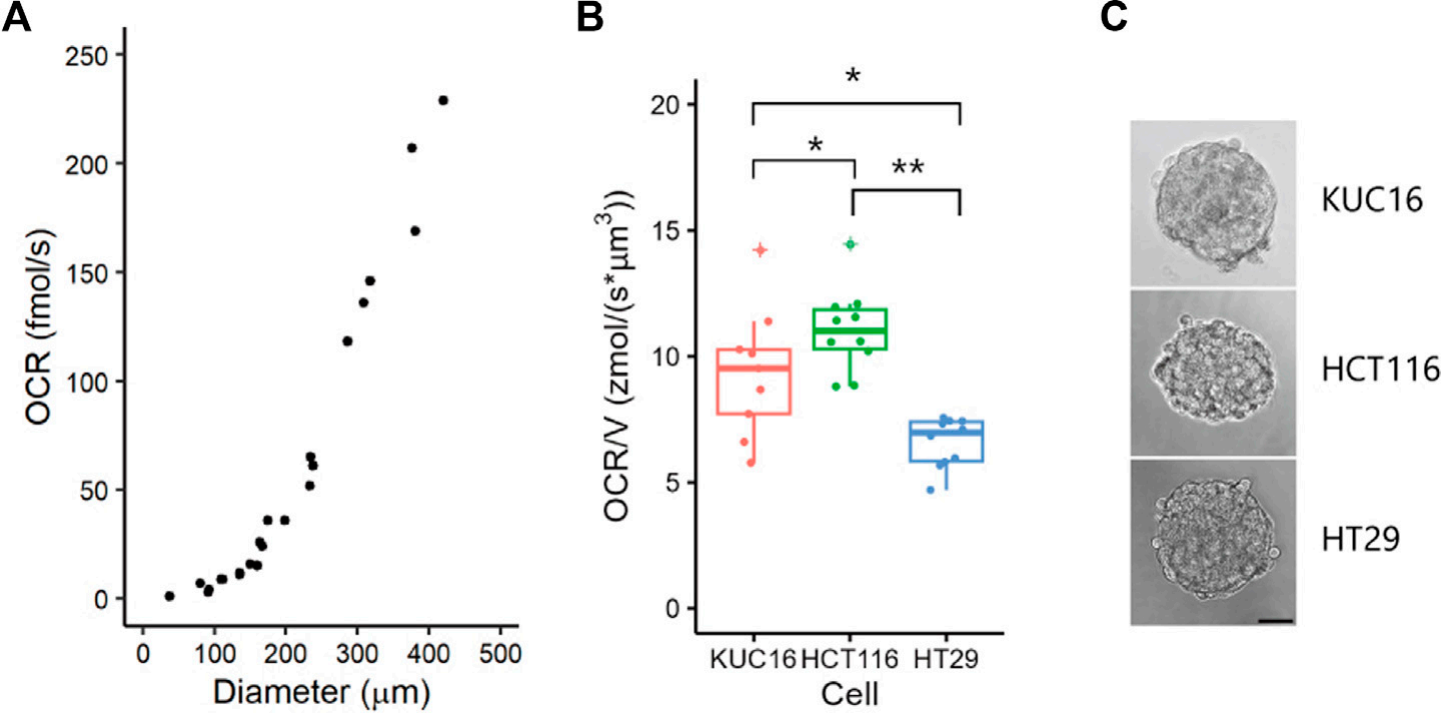
Variability of oxygen consumption rate (OCR) in electrochemical measurements. **(A)** OCRs of HCT116 spheroids of various sizes. HCT116 spheroids were cultured in suspension for 2 days. Dots represent all individual data. **(B)** Boxplots of OCR/V for KUC16, HCT116, and HT29. Dots represent all individual data and ‘+’ marks indicate outliers in boxplots. Align-and-rank data of nonparametric factorial ANOVA, *p < 0.05, **p < 0.005. Dots indicate outliers. **(C)** Bright-field images of samples. Scale bar = 50 µm.

We initially investigated the minimum sample size for which OCR could be detected in our SECM setup using HCT116 spheroids (Figure 2A). As expected, the OCR increased proportionally with the spheroid size. While we could detect signals from HCT116 spheroids with a diameter of 38 µm in SECM, for robust analysis, the minimum sample size was determined to be 100 µm in diameter to detect the difference in oxygen concentration derived from oxygen metabolism of spheroids and organoids. Therefore, for subsequent OCR measurements, unless otherwise specified, we utilised organoids with diameters ranging from 100 to 300 µm. In this size range, patient-derived cancer organoids (KUC16 and C45-4L) showed almost the same trend, exhibiting a nearly cubic relationship with diameter, indicating that organoid the oxygen metabolism was contingent upon its volume (Supplementary Figure S1). This concurs with the trend previously observed in other cancer cell lines (Mukomoto et al., 2020).

Next, we measured OCR using SECM in cancer cell lines (HCT116 and HT29) and an organoid (KUC16, Figures 2B, C). The diameters (mean ± standard deviation) of the organoids and spheroids were as follows: KUC16 = 167 ± 4 μm, HCT116 = 164 ± 6 μm, and HT29 = 173 ± 4 µm. The OCR was normalised by volume (OCR/V) to remove the size effects. The OCR/V of KUC16 was slightly lower than that of HCT116 but higher than that of HT29. KUC16 demonstrated a greater variability of OCR/V than HT29 and HCT116 cells (coefficient of variations for OCR/V, KUC16 = 0.274, HCT116 = 0.150, HT29 = 0.149). This difference in the variability of OCR/V likely reflects the differences in heterogeneity between the cell lines and organoids. The observed dissimilarities between cell lines and organoids may be attributed to variations in bioenergetic phenotypes, encompassing the production of mitochondrial and/or glycolytic ATP.

### 3.2 OCRs of patient-derived cancer organoids with different compositions

**FIGURE 3:**
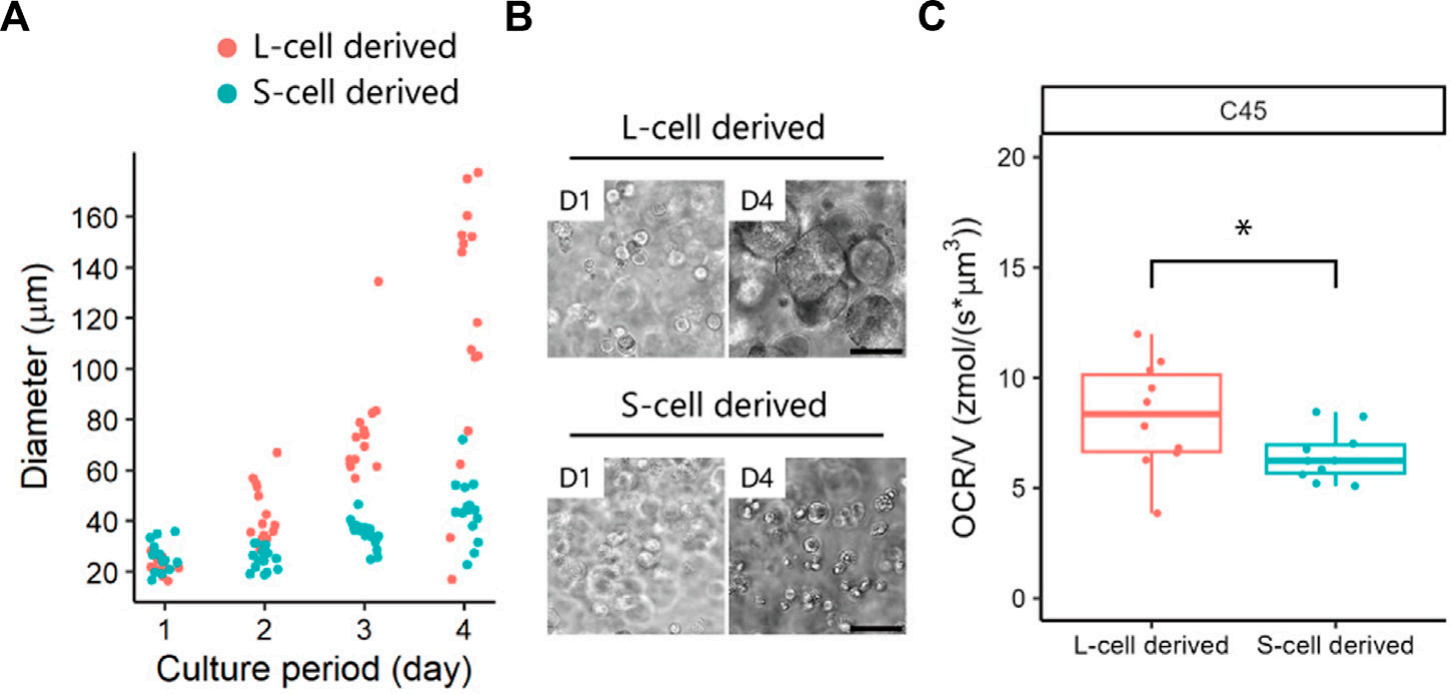
Oxygen consumption rates (OCRs) of patient-derived cancer organoids obtained from L- and S-cells. **(A)** Growth of C45 subpopulations and **(B)** phase-contrast images in 100% Matrigel. Scale bar = 100 µm. **(C)** OCR/V of organoids derived from L- and S-cells. Dots represent all individual data. Man–Whitney *U*-test, *, *p* < 0.05.

Next, we evaluated the OCR of patient-derived cancer organoids generated from different subpopulations of cells. We have previously reported the existence of subpopulations of cells with different growth abilities in CRC organoids (Coppo et al., 2023). Here, we examined tumour subpopulations from the same patient: C45-1 for S-cell-derived organoids and C45-4L for L-cell-derived organoids (Figure 1). Before analysing the oxygen metabolism, we reconfirmed that C45-4L showed a greater growth potential than C45-1 (Figures 3A, B).

The diameter (mean ± standard deviation) of the organoids was as follows: C45-1 = 196 ± 5 µm and C45-4L = 200 ± 5 µm. The differences in OCR/V between the C45 subpopulations are summarised in Figure 3C. C45-4L showed a significantly larger OCR/V and variability than C45-1, indicating the more heterogeneous nature of C45-4L. The higher OCR in C45-4L may be attributed to the presence of more cells with a higher proliferation activity. Therefore, SECM could successfully unveil the heterogeneous features of oxygen metabolism in patient-derived cancer organoids. Despite the significant differences in OCR/V between L-cell- and S-cell-derived organoids, a notable degree of overlap was detected between their values, indicating that OCR/V alone may not be sufficient to discriminate S-cells from L-cells.

As organoids derived from L-cells were composed of a mixture of L- and S-cells, the difference in their oxygen metabolism at the single-cell level remains to be elucidated. Improving the sensitivity of OCR measurements to the single-cell level is another challenge in the future. Overall, despite the limited sensitivity of our current technique at the single-cell level, we have successfully demonstrated a discernible contrast in oxygen metabolism between L-cell- and S-cell-derived organoids. Although OCR/V alone may not be sufficient to discrimination between S-cells and L-cells, our findings can provide a reference for isolating tumour subpopulations and developing novel drug screening platforms in the future. Finally, the non-invasiveness of SECM features is expected to contribute to the intact isolation of individual subpopulations without observing their growth curve, allowing for the generation of promising patient-derived organoids for use in future drug screening platforms.

## 4 Conclusion

The present study employed SECM to assess the oxygen metabolism of patient-derived organoids originating from CRC tumours and investigated whether metabolic differences were present between the subclones. SECM could detect heterogeneous oxygen metabolism in patient-derived cancer organoids, a phenomenon that was not observed in conventional cancer cell lines. Furthermore, organoids derived from L-cells exhibited a higher oxygen metabolism rate than those derived from S-cells. The current cancer therapies have been predominantly designed and developed against rapidly dividing cancer cells; therefore, the importance of quiescent or slow-growing cancer cells, such as S-cells, has been underestimated. Consequently, these slow-growing cancer cells may survive the anticancer treatment, revert to fast-growing cancer cells, and act as a reservoir for tumour regrowth. The application of SECM for non-invasive discrimination of S-cells and L-cells, coupled with our discoveries, can aid the development of a drug screening platform that targets the slow-growing subpopulation.

## Data Availability

The raw data supporting the conclusion of this article will be made available by the authors, without undue reservation.
