# Supplementary material for: Oxygen metabolism analysis of a single organoid for non-invasive discrimination of cancer subpopulations with different growth capabilities
Source: Front Bioeng Biotechnol. 2023 May 18;11:1184325. doi: 10.3389/fbioe.2023.1184325 (PMC10232988; doi:10.3389/fbioe.2023.1184325)
Supplement: Supplementary file 1 [file DataSheet1.PDF]

Supplementary Figure

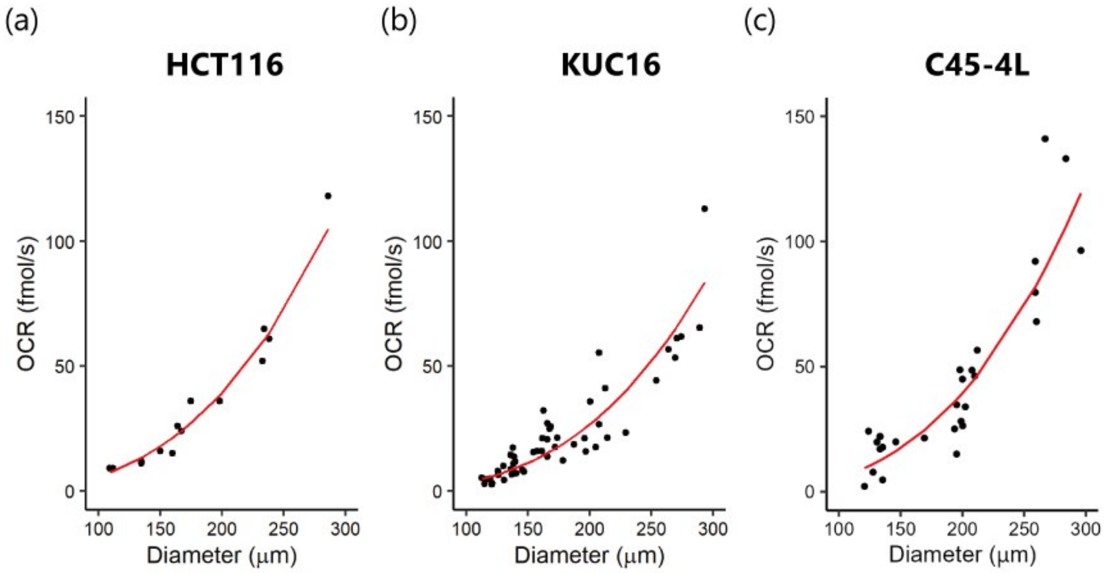

Figure S1. Oxygen consumption rates (OCRs) of HCT116 spheroids and patient-derived cancer organoids (KUC16 and C45-4L) of various sizes. Red lines represent the approximate curves determined by nonlinear regression analysis. (a) HCT116, red: $y = 1.95 \times 10^{-5} x^{2.74}$, Pearson coefficient of correlation = 0.94. (b) KUC16, red: $y = 3.52 \times 10^{-6} x^{2.99}$, Pearson coefficient of correlation = 0.89. (c) C45-4L, red: $y = 1.21 \times 10^{-5} x^{2.83}$, Pearson coefficient of correlation = 0.87.
